# *In Vivo* Stabilized SB3, an Attractive GRPR Antagonist, for Pre- and Intra-Operative Imaging for Prostate Cancer

**DOI:** 10.1007/s11307-018-1185-z

**Published:** 2018-03-19

**Authors:** Ingrid L. Bakker, Sandra T. van Tiel, Joost Haeck, Gabriela N. Doeswijk, Erik de Blois, Marcel Segbers, Theodosia Maina, Berthold A. Nock, Marion de Jong, Simone U. Dalm

**Affiliations:** 1 Department of Radiology and Nuclear Medicine, Erasmus MC, Room No. Na2510, Wytemaweg 80, 3015 CN Rotterdam, The Netherlands; 2 Molecular Radiopharmacy, INSRATES, NCSR “Demokritos”, Athens, Greece

**Keywords:** Gastrin-releasing peptide receptor, Radio-guided surgery, SPECT/MRI, NEP inhibition, Tumor imaging

## Abstract

**Purpose:**

The gastrin-releasing peptide receptor (GRPR), overexpressed on various tumor types, is an attractive target for receptor-mediated imaging and therapy. Another interesting approach would be the use of GRPR radioligands for pre-operative imaging and subsequent radio-guided surgery, with the goal to improve surgical outcome. GRPR radioligands were successfully implemented in clinical studies, especially Sarabesin 3 (SB3) is an appealing GRPR antagonist with high receptor affinity. Gallium-68 labeled SB3 has good *in vivo* stability, after labeling with Indium-111; however, the molecule shows poor *in vivo* stability, which negatively impacts tumor-targeting capacity. A novel approach to increase *in vivo* stability of radiopeptides is by co-administration of the neutral endopeptidase (NEP) inhibitor, phosphoramidon (PA). We studied *in vivo* stability and biodistribution of [^111^In]SB3 without/with (−/+) PA in mice. Furthermore, SPECT/MRI on a novel, state-of-the-art platform was performed.

**Procedures:**

GRPR affinity of SB3 was determined on PC295 xenograft sections using [^125^I]Tyr^4^-bombesin with tracer only or with increasing concentrations of SB3. For *in vivo* stability, mice were injected with 200/2000 pmol [^111^In]SB3 −/+ 300 μg PA. Blood was collected and analyzed. Biodistribution and SPECT/MRI studies were performed at 1, 4, and 24 h postinjection (p.i.) of 2.5 MBq/200 pmol or 25 MBq/200 pmol [^111^In]SB3 −/+ 300 μg PA in PC-3-xenografted mice.

**Results:**

SB3 showed high affinity for GRPR (IC_50_ 3.5 nM). Co-administration of PA resulted in twice higher intact peptide *in vivo vs* [^111^In]SB3 alone. Biodistribution studies at 1, 4, and 24 h p.i. show higher tumor uptake values with PA co-administration (19.7 ± 3.5 *vs* 10.2 ± 1.5, 17.6 ± 5.1 *vs* 8.3 ± 1.1, 6.5 ± 3.3 *vs* 3.1 ± 1.9 % ID/g tissue (*P* < 0.0001)). Tumor imaging with SPECT/MRI clearly improved after co-injection of PA.

**Conclusions:**

Co-administration of PA increased *in vivo* tumor targeting capacity of [^111^In]SB3, making this an attractive combination for GRPR-targeted tumor imaging.

## Introduction

The gastrin-releasing peptide receptor (GRPR) is a G-protein coupled receptor overexpressed in a number of cancer types such as prostate cancer, breast cancer, and gastrointestinal cancer, thereby representing an attractive molecule for tumor targeting [1, 2]. Multiple GRPR radioligands have been synthesized during the past decade, mainly for the purpose of receptor-mediated nuclear imaging and therapy [3, 4]. These studies, at the preclinical as well as clinical level, have expanded our knowledge on GRPR radioligands and have proven that targeting of GRPR for nuclear imaging and/or therapy can be beneficial in-patient care [5–8]. Moreover, research has demonstrated that GRPR antagonists are usually superior to agonists for tumor targeting, in addition to exhibiting less adverse events [9, 10].

Sarabesin 3 (SB3) is a recently developed GRPR antagonist that has excellent receptor affinity and good *in vivo* stability when labeled with the positron-emitting radionuclide gallium-68 (*t*_1/2_ = 68 min). Preclinical as well as clinical PET imaging studies using [^68^Ga]SB3 have been performed with great success [11, 12]. A novel and interesting approach would be the use of SB3 for pre-operative imaging and intra-operative radio-guided tumor detection of GRPR-expressing tumors, especially for the detection of GRPR-expressing metastases and non-palpable tumors.

**Fig. 1:**
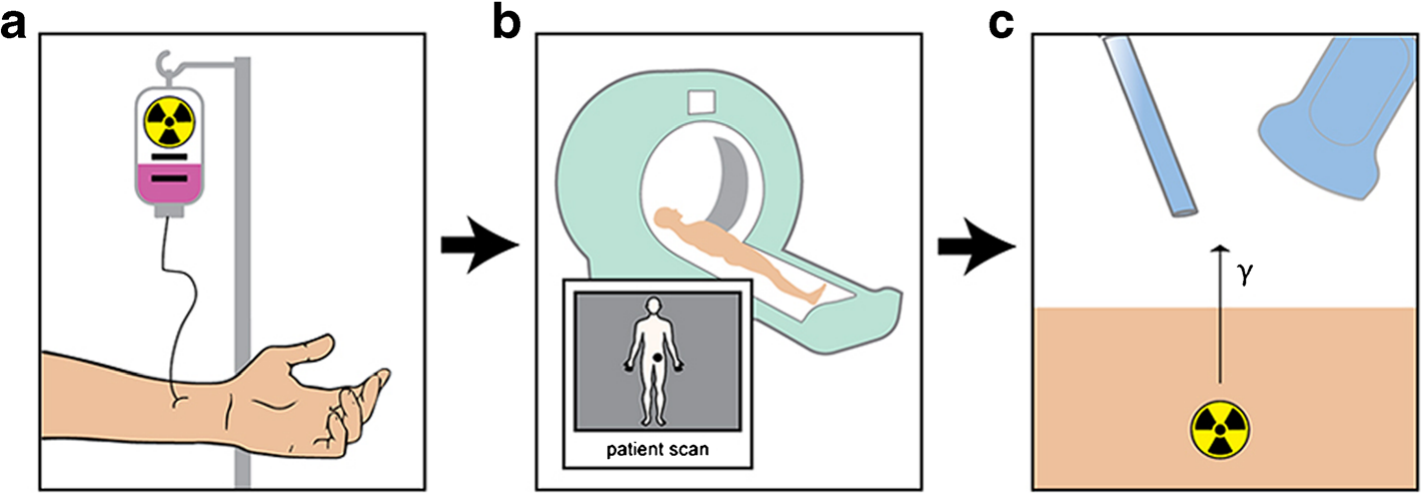
The concept of radio-guided surgery. **a** Radiopharmaceuticals directed at tumor cells are administered to the patient followed by accumulation of radioactivity in these cells over time. **b** Subsequently, full-body nuclear imaging is performed, e.g., SPECT/MRI, to localize the tumor pre-operatively. **c** Using this information, the patient is operated for removal of the tumor. Intra-operatively, a handheld device is used to detect radioactivity derived from the tumor cells to guide the surgeon towards the tumor.

Radio-guided tumor detection is based on the intra-operative localization of malignant lesions using handheld detection probes following injection of a radiopharmaceutical (Fig. 1). Combining this intra-operative method with information from pre-operative nuclear imaging by single-photon emission computed tomography (SPECT) or positron emission tomography (PET), together with computed tomography (CT) or magnetic resonance imaging (MRI), allows for accurate surgical guidance, ultimately improving surgical outcome while minimizing surgical invasiveness [13].

Currently, radio-guided surgery is successfully applied clinically for lymphoscintigraphy in melanoma and breast cancer patients, radio-guided occult lesion localization, and radio-guided seed localization of non-palpable tumors, as well as minimal invasive parathyroid surgery for removal of parathyroid adenomas [14–18]. Multiple radiopharmaceuticals and administration methods are applied for radio-guided surgery among which the application of systemically administered radiopharmaceuticals directed against targets that are overexpressed on tumor cells. Examples include ongoing preclinical and (pilot) patient studies evaluating the potential benefits of radio-guided surgery for the detection of neuroendocrine tumor and prostate cancer lesions using somatostatin receptor-targeting and prostate-specific membrane antigen-targeting radiotracers [19–24].

Ideally, for radio-guided surgery, SB3 has to be radiolabeled with a gamma emitter that has a half-life enabling detection of radioactivity prior to surgery and during the full surgical procedure. For this purpose, SB3 was therefore labeled with the clinically well-established γ-emitter indium-111 (*t*_1/2_ = 2.8 days). This [^111^In]SB3 can be used for SPECT/MRI, which offers the opportunity to combine sensitive functional imaging by SPECT with high-resolution anatomical/functional imaging by MRI. Compared to commonly used CT, MRI has a number of advantages such as improved soft tissue contrast and the added value of multi-parametric imaging, which may lead to better tumor localization.

[^111^In]SB3 has poor *in vivo* stability, ultimately resulting in low tumor uptake [25]. An explanation for the poor *in vivo* stability is degradation of the peptide by naturally occurring enzymes *in vivo*, such as the neutral endopeptidase (NEP), which cleaves peptides at the amino side of hydrophobic residues [26, 27]. To increase the *in vivo* stability of radiopeptides, multiple solutions are available, including structural modifications of a peptide or the novel approach of inhibition of enzymes responsible for enzymatic degradation. Since structural modifications are time consuming, and may have a negative impact on the binding affinity or biodistribution profile of the peptide, the latter solution is very attractive. Co-administration of the NEP inhibitor phosphoramidon (PA) enhanced the *in vivo* stability and thereby the tumor targeting capacity of multiple radiotracers [5, 26, 28–30]. The aim of our study is to evaluate whether we can also improve tumor targeting of [^111^In]SB3 by co-administration of PA to potentially use the radiotracer for pre-operative SPECT/MRI and ultimately for intra-operative radio-guided tumor detection of GRPR-expressing tumors and/or metastases as well. For this, we compared the *in vivo* stability and the biodistribution of [^111^In]SB3 without/with (−/+) PA, the latter by performing both biodistribution and SPECT/MRI studies in mice bearing GRPR-expressing PC-3 tumor xenografts.

## Materials and Methods

### Radiolabeling

**Fig. 2:**
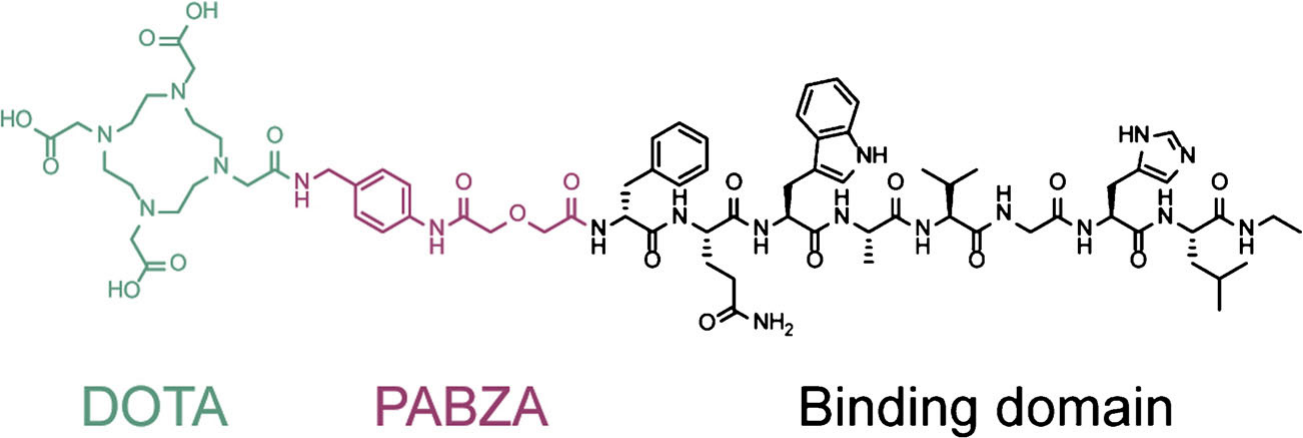
Structure of SB3.

SB3 (Fig. 2) was radiolabeled with ^111^In (Covidien) as previously described [31]. Small-volume reactions were performed in conical vials. Sodium acetate was used to control pH, resulting in a final pH value of 4–4.5 and quenchers (ascorbic and gentisic acid 3.5 mM and methionine 10 mM as final concentration) were added to prevent radiolysis. A mixture of 1 nmol SB3 and 125 MBq equivalent [^111^In]InCl_3_ and quenchers in a final volume of 140 μl were heated for 15 min at 80 °C. To complex non-incorporated In-111, after cooling down to room temperature, 5 μl of 4 mM DTPA was added. To obtain a molar activity of 12.5 MBq/nmol, after the labeling, 9 nmoles of SB3 was added. The incorporation was determined by instant thin layer chromatography (ITLC) silica gel using sodium citrate (0.1 M, pH 5) as solvent [32] and radiochemical purity (RPC) was measured by a HPLC system (Breeze, Waters), containing a 2690 quaternary pump, and radioactivity was monitored with a NaI detector, digital multichannel analyzer, and dedicated software (MetorX B.V). A Symmetry C_18_ column (5 μm × 4.6 mm × 250 mm, Waters) was used with a gradient profile of 0–5 min 100 % A, 5–5.01 min 60 % B, 5.01–20 min 80 % B, 20–20.01 min 100 % B, 20.01–25 min 100 % B, and 25.01–30 min (flow 1 ml/min), where A was 0.1 % trifluoroacetic acid (TFA) in H_2_O and B was LC-MS grade methanol. No further purification was required.

A molar activity of 12.5 MBq/nmol and 125 MBq/nmol was used for the biodistribution and SPECT/MRI studies, respectively. For the *in vivo* stability studies, both molar activities were used. To determine the affinity of SB3 for the human GRPR in a competition-binding assay, Tyr^4^-Bombesin (Sigma-Aldrich) was radiolabeled with iodine-125 based on the Iodo Beads® (Pierce) method, adapted with 5-min pre-incubation before addition of the peptide, as previously described [33, 34]. In short, 100 μl of PBS 50 mM Pi pH 7.5, sodium iodide solution (10–20 MBq Na[^125^I]I in 0.01 M NaOH), and pre-rinsed Iodo Beads® were pre-incubated for 5 min, and Iodo Beads® were removed. Then, total volume was added to 70 nmol of peptide in PBS, and the mixture was allowed to react for 5 min at room temperature. To exclude non-labeled iodine, purification was performed by solid phase extraction using a J.T. Baker C_18_ cartridge followed by HPLC purification [34].

### Internalization Studies

Internalization with [^111^In]SB3 was performed in PC-3 cells, a commonly used human-derived androgen independent GRPR-expressing PC cell line (AACR). The cells were seeded in six-well plates at a density of 0.6 × 10^6^ cells per well and cultured overnight. On the day of the experiment, cells were washed twice with 37 °C PBS. Fresh internalization medium, RPMI + Glutamax with 20 mM HEPES, and 1 % bovine serum albumin fraction V pH 7.4, 10^−9^ M [^111^In]SB3 was added. The cells were incubated at 37 °C in triplicates for 60 min, and incubation was stopped by removal of the medium and rinsing with cold PBS twice. Thereafter, the cells were incubated for 10 min with acid wash buffer (50 mM Glycin-HCl/100 mM NaCl, pH 2.8). The supernatant was collected (membrane fraction); the cells were rinsed with acid wash buffer and collected in the same tube. Cells were lysed with 0.1 M NaOH and collected in another tube (internalized cell fraction). The amount of activity in the membrane and cell fraction was measured on an automatic gamma counter.

### Animal Model

Healthy or xenografted Balb c nu/nu male mice (Janvier) were used in this study. To obtain xenografted mice, animals were injected subcutaneously in the right shoulder with PC-3, 3 × 10^6^ cells in Matrigel (total volume 150 μl, solution 66 % RPMI, 33 % Matrigel) per animal. Prior to tumor cell inoculation, the PC-3 cells were cultured in RPMI medium supplemented with 10 % fetal bovine serum, 5000 IU/ml penicillin, and 5000 μg/ml streptomycin at 37 °C in a humidified atmosphere containing 5 % CO_2_. PC295 is an androgen-dependent, patient-derived cell line with high GRPR expression. Xenografts were generated by subcutaneous implantation of the PC295 human prostate tumor as previously described and were stored at − 80 °C until further use [35]. All culture supplies were purchased at Gibco.

### Competition Binding Assay

For the competition binding assay, fresh frozen PC295 xenograft tissue slices (10 μm) were incubated in triplicate with 80 μl 5 × 10^−10^ M [^125^I]Tyr^4^-Bombesin, with tracer only or with an increasing concentration (10^−12^ to 10^−6^ M) of unlabeled SB3 for 1 h and exposed to super resolution phosphor screens (PerkinElmer) for 48 h. The phosphor screens were read using the Cyclone (PerkinElmer) and quantified using OptiQuant software. Binding of unblocked [^125^I]Tyr^4^-bombesin to the tissue slices was set at 100 %, and the percentage of binding relative to the unblocked tissue sample was calculated for the blocked tissue sections. These values were used to determine the IC_50_ value of unlabeled SB3.

### Stability Studies

To determine the stability of [^111^In]SB3 −/+ PA *in vivo*, healthy mice (*n* = 3 per group) were intravenously injected with 200 pmol/25 MBq or 2000 pmol/25 MBq [^111^In]SB3 (volume 100 μl), co-injected with either 300 μg/100 μl PA (Peptides International Inc.) in 0.5 % ethanol or 100 μl saline solution. Five minutes after injection of [^111^In]SB3 −/+ PA, blood (0.5–1 ml) was collected by cardiac puncture, while animals were under isoflurane/O_2_ anesthesia, and transferred to blood-collection tubes containing 90 USP units of lithium heparin (spray coated), HPLC grade ethanol was added in a 1:1 (*v*/*v*) ratio, and the tubes were placed on ice. Blood samples were centrifuged 5 min at 1250*g*, and supernatant was collected. Next, the collected supernatant was centrifuged again for 5 min at 12,500*g*. The secondary supernatant was collected and diluted with MilliQ down to < 25 % ethanol before injection on HPLC. The samples were analyzed by HPLC (Alliance, Waters) including a Symmetry C_18_ column (5 μm × 4.6 mm × 250 mm). Elution was performed in a 1-ml/min flow, completed in 30 min, with the gradient 0–5 min 100 % A, 5–5.01 min 40 % A 60 % B, 5.01–20 min 20 % A 80 % B, 20–20.01 min 100 % B, 20.01–25 min 100 % B, and 25.01–30 min 100 % A, where A was 0.1 % TFA in H_2_O and B was methanol. In one animal, the sample volume was too low for further analysis; this animal was therefore excluded.

### Biodistribution Studies

Tumor-bearing mice received an intravenous injection of 200 pmol/2.5 MBq [^111^In]SB3, co-injected with either 300 μg/100 μl PA in 0.5 % ethanol or 100 μl saline solution (*n* = 4 animals per group for each time point). In previous *in vivo* studies, 200 pmol appeared to be the optimal peptide amount (regarding uptake in tumor and pancreas; data not shown). To determine specificity of tumor and organ uptake, blocking experiments were performed; an additional group of animals (*n* = 3) was pre-injected with an excess (20 nmol) of unlabeled SB3 followed by injection of the radiotracer −/+ PA. At three different time points p.i. (1, 4, and 24 h), animals were euthanized and blood, organs, and tumors were collected, weighed, and measured in a γ-counter (1480 WIZARD automatic γ-counter, PerkinElmer). For γ-counter measurements, an isotope specific energy window, a counting time of 60 s, and a counting error ≤ 5 % were used. Results were expressed as the percentage of the injected dose per gram tissue (% ID/g tissue). Animals that had ≥ 7 % ID in the tail were excluded.

### SPECT/MRI

For SPECT/MRI, PC-3-xenografted mice were intravenously injected with 25 MBq/200 pmol [^111^In]SB3, co-injected with either 300 μg/100 μl PA in 0.5 % ethanol or 100 μl saline solution. Subsequently, imaging was performed 1, 4, and 24 h p.i. of the radiotracer, while animals were anesthetized using isoflurane/O_2_, body temperature was maintained using warm air flow, and respiratory rate was monitored. Whole-body SPECT images were acquired using the novel and state-of-the-art four-head multipinhole system (NanoScan SPECT/MRI, Mediso Medical Imaging) in 30 min (28 projections, 29 s/projection, and 7-cm axial field of view). Images were reconstructed using OSEM with six iterations and four subsets on a 120 × 120 matrix with 0.25 × 0.25 × 0.25-mm isotropic voxels. MR-based attenuation correction was applied to all images. T2 MR images were acquired using a spin echo sequence (TR/TE = 4500/52 ms) with a 35-mm-diameter solenoid coil. Other scan parameters were field of view 70 mm, matrix 256 × 128, and slice thickness 1 mm with 0.1-mm spacing between slices. An ROI of the tumor was drawn by an experienced technician on the T2-weighted MR image to measure the intensity in the SPECT image.

### Statistics

All statistical evaluations were performed with GraphPad Prism software (version 5.01). The IC_50_ value was calculated using a log (inhibitor) *vs* response model. To compare *in vivo* stability −/+ PA, radioactivity uptake in the tumor −/+ PA and radioactivity uptake in the tumor without and with an excess of unlabeled peptide an unpaired *t* test was used. *P* values < 0.05 were considered statistically significant. Tumor to organ uptake ratios were calculated per animal and expressed as mean and standard deviation per group.

## Results

### Radiolabeling

The radiochemical purity and radiometal incorporation were both > 90 %.

### Internalization Studies

In internalization experiments with [^111^In]SB3 in PC-3 cells, the membrane fraction contained almost four times more radioactivity compared to the cell fraction.

### Competition Binding Assay

**Fig. 3:**
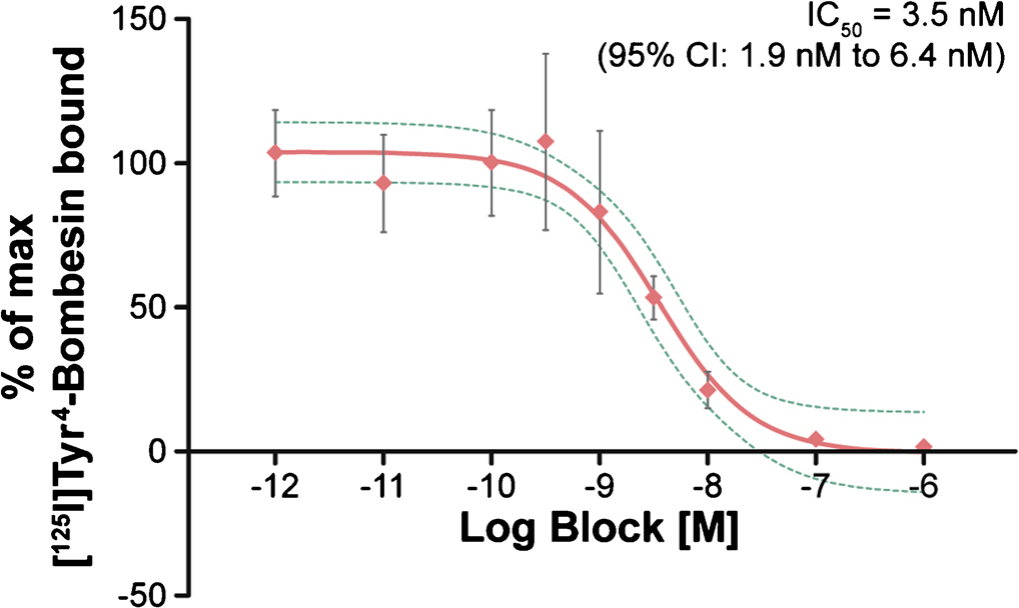
Competitive binding assay using [^125^I]Tyr^4^-bombesin blocked with increasing concentrations of unlabeled SB3. The 95 % CI is shown in green.

Quantified binding of [^125^I]Tyr^4^-bombesin to PC259 tissue sections without and with increasing concentrations of unlabeled SB3 resulted in an IC_50_ value of 3.5 nM (95 % CI 1.9 to 6.4 nM) (Fig. 3).

### Stability Studies

**Fig. 4:**
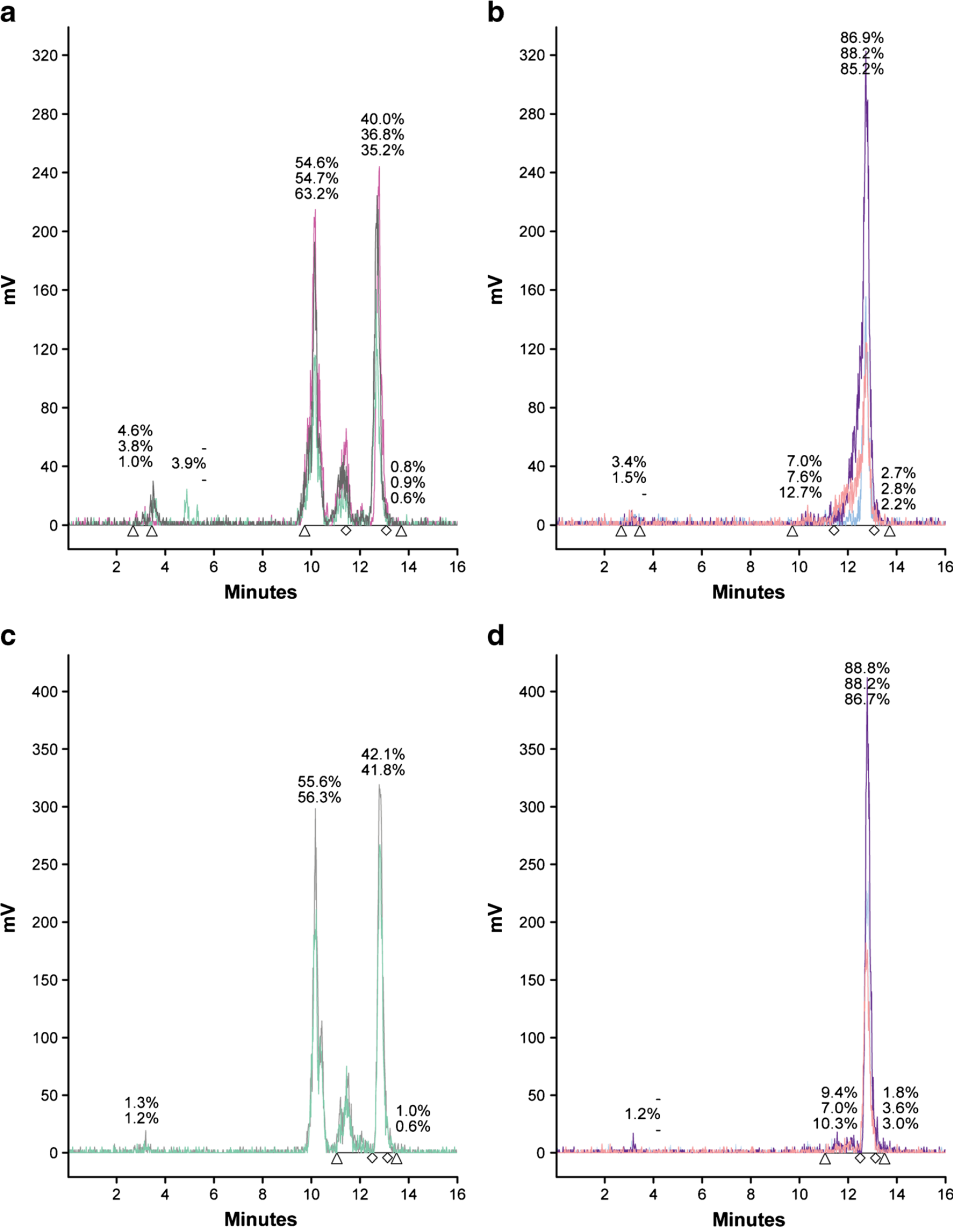
*In vivo* stability of [^111^In]SB3. HPLC analysis of extracted radioactivity from blood 5 min p.i. Of a low peptide amount (200 pmol/25 MBq) either **a** in the absence of PA or **b** in the presence of PA, and 5 min p.i. With a high peptide amount (2000 pmol/25 MBq) either **c** in the absence or **d** in the presence of PA. *-* peak is missing.

*In vivo* stability of [^111^In]SB3 −/+ PA was analyzed in blood samples, collected 5 min p.i. In the animals receiving 200 pmol [^111^In]SB3, 37.3 ± 2.5 % of the remaining circulating activity consisted of intact radiotracer, while this value significantly increased (*P* < 0.0001) to 86.7 ± 1.5 % when [^111^In]SB3 was co-injected with PA (Fig. 4a, b). When a 10× higher peptide amount was administered, these values were 42.0 ± 0.2 % *vs* 87.9 ± 1.1 % (*P* < 0.0001) of the radiotracer still being intact in the blood, respectively (Fig. 4c, d), indicating a clear increase of stability even with 2000-pmol peptide.

### Biodistribution Studies

**Fig. 5:**
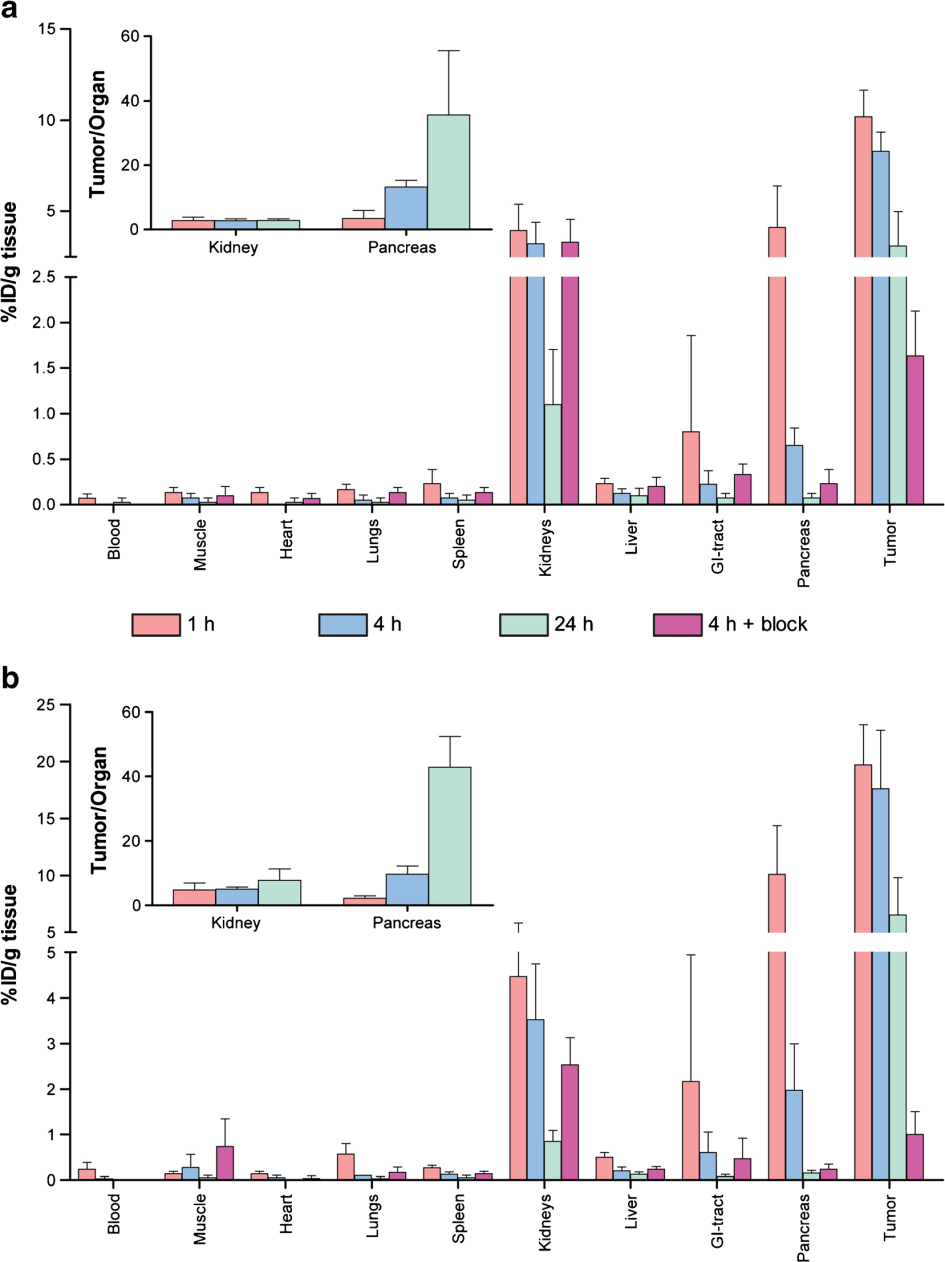
Blood, organ, and tumor uptake of [^111^In]SB3 **a** administered without PA and **b** with PA. Tumor to kidney and tumor to pancreas ratios are displayed in the insets of graphs of **a** and **b**.

The results of the biodistribution studies are displayed in Fig. 5. A maximum tumor uptake of 10.2 ± 1.5 % ID/g tissue was reached using 2.5 MBq/200 pmol [^111^In]SB3 1 h p.i. in contrast to 19.7 ± 3.5 % ID/g tissue after injection of [^111^In]SB3 + PA. At all studied time points, there was significantly higher (*P* < 0.0001) tumor uptake when PA was co-administered with [^111^In]SB3. Next to tumor uptake, high radiotracer uptake was also observed in the GRPR-expressing pancreas and in the kidneys, the latter as a result of renal excretion and partial reabsorption of the radiopeptide. The increased *in vivo* stability of [^111^In]SB3 + PA also resulted in an increase in pancreatic uptake (4.1 ± 2.3 *vs* 10.1 ± 4.3 % ID/g tissue 1 h p.i. −/+ PA, respectively), while renal uptake remained similar (3.9 ± 1.4 *vs* 4.5 ± 1.4 % ID/g tissue 1 h p.i. −/+ PA, respectively). At 4 and 24 h p.i., the same pattern was observed. A similar tumor to pancreas radioactivity ratio was found for [^111^In]SB3 −/+ PA (3.4 ± 2.5 *vs* 2.2 ± 0.8, 1 h p.i. −/+ PA, respectively). The tumor to kidney ratio was more favorable however when the radiotracer was combined with PA (2.8 ± 1.0 *vs* 4.8 ± 2.2, 1 h p.i. −/+ PA, respectively). Also, radiotracer uptake in the pancreas and the tumor was receptor-specific as can be concluded from the significantly decreased (*P* ≤ 0.001) uptake when an excess of unlabeled SB3 was co-administered.

### SPECT/MRI

**Fig. 6:**
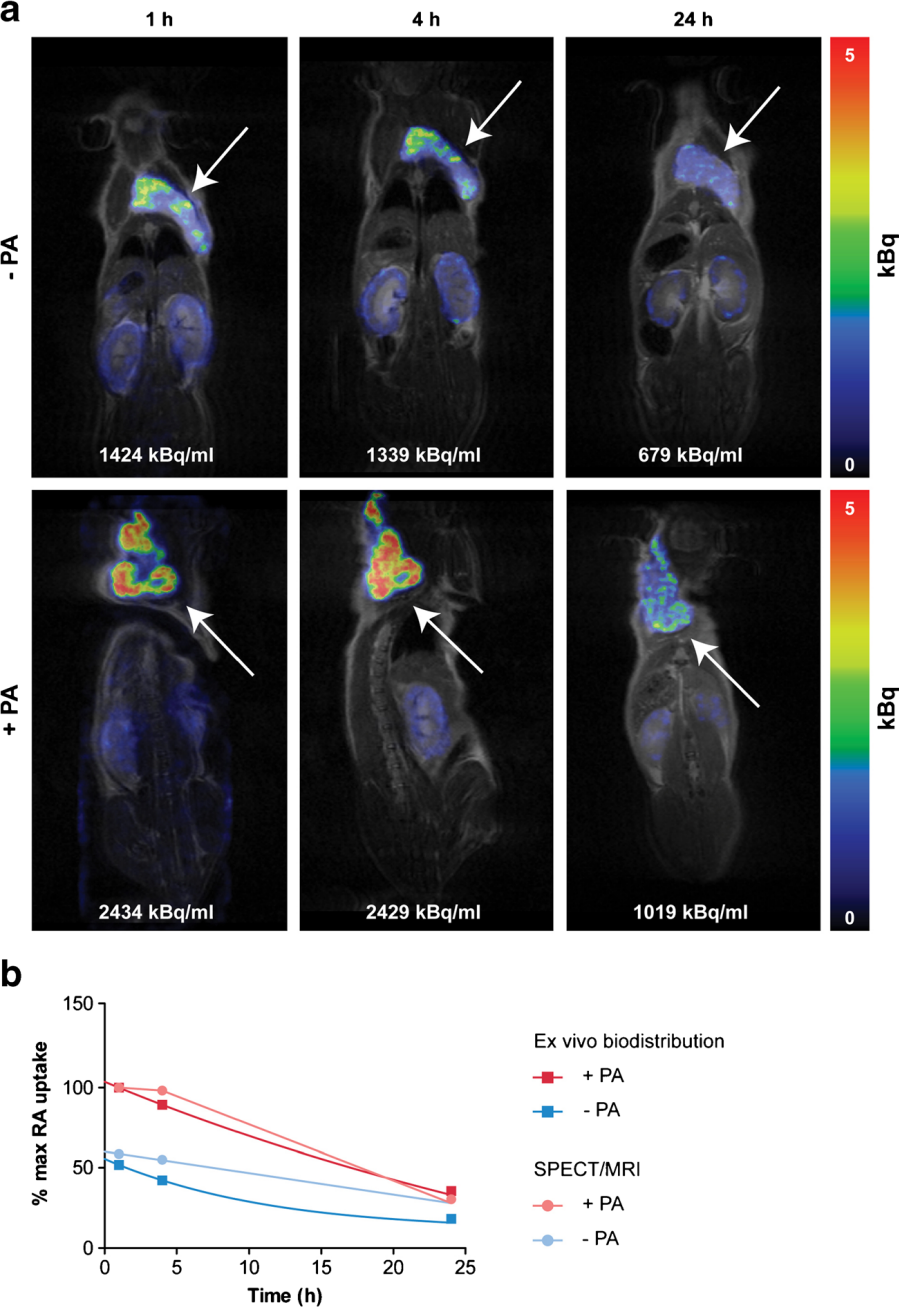
**a** SPECT/MRI 25 MBq/200 pmol [^111^In]SB3 without (top panel) and with (bottom panel) co-administration of PA. The arrows indicate the tumor. **b** Time activity curve of radioactivity uptake in PC-3 xenografts obtained by *ex vivo* biodistribution (200 pmol/2.5 MBq/mouse) and by ROI analysis on SPECT/MRI (200 pmol/25 MBq/mouse).

In line with the biodistribution studies, SPECT/MR imaging performed 1, 4, and 24 h p.i. of 25 MBq/200 pmol [^111^In]SB3 −/+ PA resulted in clearly improved tumor visualization when the radiotracer was combined with PA, at all imaging time points (Fig. 6a). ROI analysis showed an increase of 70 % in signal intensity (1424 *vs* 2434 kBq/ml) 1 h p.i. of [^111^In]SB3 only and [^111^In]SB3 + PA, respectively. Intensity curves and the biodistribution time activity curves were normalized to 100 % at 1 h for the + PA group. The normalized curves obtained from SPECT/MR images and biodistribution studies showed a well-comparable pattern for the tumor radioactivity (Fig. 6b).

## Discussion

The GRPR is overexpressed at high incidence and high density on various tumors, including prostate cancer, making it an appealing molecule for tumor targeting [2]. Over time, multiple and promising GRPR targeting radiotracers have been developed, mostly for nuclear imaging and therapeutic purposes. Another interesting approach would be the application of GRPR radioligands for radio-guided surgery since handheld probes and cameras can be used to detect radioactive decay intra-operatively. For GRPR targeting to be successful, the radiotracer applied should have good *in vivo* stability, good receptor affinity, and favorable pharmacokinetic properties.

In this study, we evaluated whether co-administration of the NEP inhibitor PA with [^111^In]SB3, a radiolabeled GRPR antagonist, leading to *in vivo* stabilization of the radioligand, would improve the application use of the radiopharmaceutical for pre-operative SPECT/MRI, and the future use of peri-operative scintigraphy and radio-guided surgery. Currently, MRI is the most powerful imaging tool for prostate cancer, among other factors because of its high soft-tissue contrast and high resolution [36, 37]. Combining MRI with sensitive and specific targeted nuclear imaging by SPECT potentially improves tumor detection.

Thereto, we performed *in vivo* stability studies, biodistribution studies and SPECT/MR imaging in healthy mice and mice subcutaneously xenografted with the GRPR-expressing prostate cancer cell line PC-3. Prior to this, we confirmed a considerable higher membrane binding compared to internalized fraction of [^111^In]SB3 as expected for a receptor antagonist. Also, we determined the receptor affinity of the unlabeled compound on tissue slices of xenografts generated from the human prostate tumor PC295, confirming previous reported affinities of unlabeled SB3 and ^nat^In labeled SB3 in the low nanomolar range [25]. This data showed that SB3 had excellent affinity for the GRPR, confirming previous findings [11].

*In vivo* stability studies 5 min p.i. of [^111^In]SB3 −/+ PA in healthy mice showed twice as much intact radiopeptide in the blood when PA was co-administered, similar to what has been reported for other radiopeptides [5, 28, 29]. The large amount of unlabeled compound might hamper formation and detection of radiometabolites, so caution is advised when comparing the results of the low and high peptide amounts. However, even when using a high amount of peptide, a clear effect of PA was still observed.

The metabolic susceptibility of peptides can be influenced by a multitude of characteristics. Next to the peptide structure, also charge and configuration can play a role. The most well-known approach to protect peptides against degradation is modify the peptide, e.g., by stabilizing peptide bonds. It was found that specific changes of the C-terminal structure of peptide gastrin-releasing peptide analogues resulted in receptor antagonists with improved potency and stability [38]. Previously, it has been reported that charge, peptide chain length, choice of linker, etc., can all have large effects on metabolic stability and pharmacokinetics of GRP ligands [39, 40].

Concerning the biodistribution studies, stabilization of [^111^In]SB3 by inhibition of NEP resulted in higher tumor uptake. Next to higher tumor uptake, high uptake of the radiotracer was also observed in the pancreas. Even though the radiopeptide uptake in the pancreas increased when [^111^In]SB3 was co-administered with PA, the tumor to pancreas ratio remained unaffected. Nevertheless, this high uptake in the pancreas should be kept in mind when the radiotracer would be used for radio-guided surgery of lesions close to the pancreas. In this case, the surgeon should carefully evaluate whether the signal measured by a gamma detection probe and/or a handheld gamma camera is derived from tumor lesions or neighboring healthy tissue. However, as can be seen from the biodistribution data, radioactivity washout from the pancreas was relatively fast, while tumor radioactivity uptake declined at a lower rate. This was also observed in previously published studies [11, 41–43]. So, determining the time window with optimal tumor/pancreas ratio is required for optimal pre-operative and intra-operative tumor detection. In this respect, the uptake in the kidneys and the bladder due to renal excretion of the radiotracer should also be taken into account.

An interesting next step would be to modify the peptide to create a molecule that can serve both as radioactivity and fluorescence carrier. Even though radio-guided surgery is profitable, it has limitations. So, the use of radiotracers allows for gross tumor localization by detection of γ-photons outside of the body, but this method is not suited for accurate tumor delineation during surgery [44]. On the other hand, fluorescence imaging is limited by the penetration depth of fluorescent light, which can only be detected when at close distance. However, fluorescence imaging does allow exact tumor delineation and can provide real-time intra-operative visualization [44]. Combining the strengths of radio-guided and fluorescence guided surgery can therefore be of benefit.

SPECT/MR imaging resulted in successful identification of tumor lesions on scans and, as expected, higher radioactivity uptake was visualized when [^111^In]SB3 was co-administered with PA. Time activity curves obtained with *ex vivo* biodistribution studies and ROI analysis on SPECT/MR images demonstrated similar patterns, indicating the SPECT/MRI platform to correctly depict the pharmacokinetics of the radiotracer.

With respect to the commercially available NEP inhibitor PA, to date, pre-clinical studies evaluating its potential in potentiating the use of radiotracers have not indicated any adverse events [5, 28]. For application in patients, NEP inhibitors that are already approved for clinical use such as Sacubitril and Racecadotril might be applied [45].

Furthermore, depending on the cancer type for which [^111^In]SB3 + PA will be used for tumor detection pre- and intra-operatively, the application should be compared to that using other radiotracers under investigation or applied for similar purposes, e.g., lymphoscintigraphy for sentinel lymph node biopsy in breast cancer patients and PSMA radio-guided surgery in prostate cancer patients [21, 46].

## Conclusions

Co-administration of [^111^In]SB3 with the NEP inhibitor PA led to a significant increase of *in vivo* stability of [^111^In]SB3. So, SPECT/MRI using [^111^In]SB3 + PA resulted in excellent visualization of tumors in our preclinical model, indicating its potency for (pre-operative) imaging and future radio-guided surgery applications.

## References

[1] Jensen RT, Battey JF, Spindel ER, Benya RV (2008). International Union of Pharmacology. LXVIII. Mammalian bombesin receptors: nomenclature, distribution, pharmacology, signaling, and functions in normal and disease states. Pharmacol Rev.

[2] Reubi JC, Wenger S, Schmuckli-Maurer J, Schaer JC, Gugger M (2002). Bombesin receptor subtypes in human cancers: detection with the universal radioligand ^125^I-[D-TYR(6), beta-ALA(11), PHE(13), NLE(14)] bombesin(6-14). Clin Cancer Res.

[3] Sancho V, Di Florio A, Moody TW, Jensen RT (2011). Bombesin receptor-mediated imaging and cytotoxicity: review and current status. Curr Drug Deliv.

[4] Moreno P, Ramos-Alvarez I, Moody TW, Jensen RT (2016). Bombesin related peptides/receptors and their promising therapeutic roles in cancer imaging, targeting and treatment. Expert Opin Ther Targets.

[5] Chatalic KL, Konijnenberg M, Nonnekens J (2016). In vivo stabilization of a gastrin-releasing peptide receptor antagonist enhances PET imaging and radionuclide therapy of prostate cancer in preclinical studies. Theranostics.

[6] Dumont RA, Tamma M, Braun F, Borkowski S, Reubi JC, Maecke H, Weber WA, Mansi R (2013). Targeted radiotherapy of prostate cancer with a gastrin-releasing peptide receptor antagonist is effective as monotherapy and in combination with rapamycin. J Nucl Med.

[7] Nock BA, Kaloudi A, Lymperis E, Giarika A, Kulkarni HR, Klette I, Singh A, Krenning EP, de Jong M, Maina T, Baum RP (2017). Theranostic perspectives in prostate cancer with the gastrin-releasing peptide receptor antagonist NeoBOMB1: preclinical and first clinical results. J Nucl Med.

[8] Stoykow C, Erbes T, Maecke HR, Bulla S, Bartholomä M, Mayer S, Drendel V, Bronsert P, Werner M, Gitsch G, Weber WA, Stickeler E, Meyer PT (2016). Gastrin-releasing peptide receptor imaging in breast cancer using the receptor antagonist ^68^Ga-RM2 and PET. Theranostics.

[9] Bodei L, Ferrari M, Nunn A (2007). ^177^Lu-AMBA bombesin analogue in hormone refractory prostate cancer patients: a phase I escalation study with single-cycle administrations [abstract]. Eur J Nucl Med Mol Imaging.

[10] Cescato R, Maina T, Nock B, Nikolopoulou A, Charalambidis D, Piccand V, Reubi JC (2008). Bombesin receptor antagonists may be preferable to agonists for tumor targeting. J Nucl Med.

[11] Maina T, Bergsma H, Kulkarni HR, Mueller D, Charalambidis D, Krenning EP, Nock BA, de Jong M, Baum RP (2016). Preclinical and first clinical experience with the gastrin-releasing peptide receptor-antagonist [^68^Ga]SB3 and PET/CT. Eur J Nucl Med Mol Imaging.

[12] Bakker I, Fröberg AC, Busstra MB (2016). PET imaging of prostate cancer using the GRPr-targeting ligand Sarabesin 3 prior to radical prostatectomy [abstract]. Eur J Nucl Med Mol Imaging.

[13] Povoski SP, Neff RL, Mojzisik CM (2009). A comprehensive overview of radioguided surgery using gamma detection probe technology. World J Surg Oncol.

[14] Bluemel C, Herrmann K, Giammarile F, Nieweg OE, Dubreuil J, Testori A, Audisio RA, Zoras O, Lassmann M, Chakera AH, Uren R, Chondrogiannis S, Colletti PM, Rubello D (2015). EANM practice guidelines for lymphoscintigraphy and sentinel lymph node biopsy in melanoma. Eur J Nucl Med Mol Imaging.

[15] Giammarile F, Bozkurt MF, Cibula D, Pahisa J, Oyen WJ, Paredes P, Olmos RV, Sicart SV (2014). The EANM clinical and technical guidelines for lymphoscintigraphy and sentinel node localization in gynaecological cancers. Eur J Nucl Med Mol Imaging.

[16] Valdes Olmos RA, Vidal-Sicart S, Manca G (2017). Advances in radioguided surgery in oncology. Q J Nucl Med Mol Imaging.

[17] Desiato V, Melis M, Amato B (2016). Minimally invasive radioguided parathyroid surgery: a literature review. Int J Surg.

[18] Chan BK, Wiseberg-Firtell JA, Jois RH (2015). Localization techniques for guided surgical excision of non-palpable breast lesions. Cochrane Database Syst Rev.

[19] Cuccurullo V, Di Stasio GD, Mansi L (2017). Radioguided surgery with radiolabeled somatostatin analogs: not only in GEP-NETs. Nucl Med Rev Cent East Eur.

[20] Adams S, Baum RP (2000). Intraoperative use of gamma-detecting probes to localize neuroendocrine tumors. Q J Nucl Med.

[21] Rauscher I, Eiber M, Maurer T (2017). [PSMA-radioguided surgery for salvage lymphadenectomy in recurrent prostate cancer] ^111^In-PSMA-radioguided surgery beim oligometastasierten Prostatakarzinomrezidiv. Aktuelle Urol.

[22] Robu S, Schottelius M, Eiber M, Maurer T, Gschwend J, Schwaiger M, Wester HJ (2017). Preclinical evaluation and first patient application of ^99m^Tc-PSMA-I&S for SPECT imaging and radioguided surgery in prostate cancer. J Nucl Med.

[23] Schottelius M, Wirtz M, Eiber M, Maurer T, Wester HJ (2015). [^111^In]PSMA-I&T: expanding the spectrum of PSMA-I&T applications towards SPECT and radioguided surgery. EJNMMI Res.

[24] Maurer T, Weirich G, Schottelius M, Weineisen M, Frisch B, Okur A, Kübler H, Thalgott M, Navab N, Schwaiger M, Wester HJ, Gschwend JE, Eiber M (2015). Prostate-specific membrane antigen-radioguided surgery for metastatic lymph nodes in prostate cancer. Eur Urol.

[25] Lymperis E, Maina-Nock T, Kaloudi A (2014). Transient in vivo NEP inhibition enhances the theranostic potential of the new GRPR-antagonist [^111^In/177Lu]SB3 [abstract]. Eur J Nucl Med.

[26] Nock BA, Maina T, Krenning EP, de Jong M (2014). “To serve and protect”: enzyme inhibitors as radiopeptide escorts promote tumor targeting. J Nucl Med.

[27] Roques BP, Noble F, Dauge V (1993). Neutral endopeptidase 24.11: structure, inhibition, and experimental and clinical pharmacology. Pharmacol Rev.

[28] Marsouvanidis PJ, Melis M, de Blois E, Breeman WAP, Krenning EP, Maina T, Nock BA, de Jong M (2014). In vivo enzyme inhibition improves the targeting of [^177^Lu]DOTA-GRP(13-27) in GRPR-positive tumors in mice. Cancer Biother Radiopharm.

[29] Maina T, Kaloudi A, Valverde IE, Mindt TL, Nock BA (2017). Amide-to-triazole switch vs. in vivo NEP-inhibition approaches to promote radiopeptide targeting of GRPR-positive tumors. Nucl Med Biol.

[30] Suda H, Aoyagi T, Takeuchi T, Umezawa H (1973). Letter: a thermolysin inhibitor produced by *Actinomycetes*: phospholamidon. J Antibiot (Tokyo).

[31] de Blois E, Chan HS, Konijnenberg M, de Zanger R, Breeman WA (2012). Effectiveness of quenchers to reduce radiolysis of ^111^In- or ^177^Lu-labelled methionine-containing regulatory peptides. Maintaining radiochemical purity as measured by HPLC. Curr Top Med Chem.

[32] Bakker WH, Albert R, Bruns C, Breeman WAP, Hofland LJ, Marbach P, Pless J, Pralet D, Stolz B, Koper JW, Lamberts SWJ, Visser TJ, Krenning EP (1991). [^111^In-DTPA-D-Phe1]-octreotide, a potential radiopharmaceutical for imaging of somatostatin receptor-positive tumors: synthesis, radiolabeling and in vitro validation. Life Sci.

[33] Bakker WH, Krenning EP, Breeman WA, Koper JW, Kooij PP, Reubi JC, Klijn JG, Visser TJ, Docter R, Lamberts SW (1990). Receptor scintigraphy with a radioiodinated somatostatin analogue: radiolabeling, purification, biologic activity, and in vivo application in animals. J Nucl Med.

[34] de Blois E, Chan HS, Breeman WA (2012). Iodination and stability of somatostatin analogues: comparison of iodination techniques. A practical overview. Curr Top Med Chem.

[35] van Weerden WM, de Ridder CM, Verdaasdonk CL, Romijn JC, van der Kwast T, Schröder FH, van Steenbrugge G (1996). Development of seven new human prostate tumor xenograft models and their histopathological characterization. Am J Pathol.

[36] Guneyli S, Erdem CZ, Erdem LO (2016). Magnetic resonance imaging of prostate cancer. Clin Imaging.

[37] Futterer JJ, Briganti A, De Visschere P (2015). Can clinically significant prostate cancer be detected with multiparametric magnetic resonance imaging? A systematic review of the literature. Eur Urol.

[38] Heimbrook DC, Saari WS, Balishin NL, Fisher TW, Friedman A, Kiefer DM, Rotberg NS, Wallen JW, Oliff A (1991). Gastrin releasing peptide antagonists with improved potency and stability. J Med Chem.

[39] Marsouvanidis PJ, Maina T, Sallegger W, Krenning EP, de Jong M, Nock BA (2013). Tumor diagnosis with new ^111^In-radioligands based on truncated human gastrin releasing peptide sequences: synthesis and preclinical comparison. J Med Chem.

[40] Kaloudi A, Nock BA, Lymperis E, Krenning EP, de Jong M, Maina T (2016). ^99m^Tc-labeled gastrins of varying peptide chain length: distinct impact of NEP/ACE-inhibition on stability and tumor uptake in mice. Nucl Med Biol.

[41] Dalm SU, Bakker IL, de Blois E, Doeswijk GN, Konijnenberg MW, Orlandi F, Barbato D, Tedesco M, Maina T, Nock BA, de Jong M (2017). ^68^Ga/^177^Lu-NeoBOMB1, a novel radiolabeled GRPR antagonist for theranostic use in oncology. J Nucl Med.

[42] Mansour N, Paquette M, Ait-Mohand S, Dumulon-Perreault V, Guerin B (2018). Evaluation of a novel GRPR antagonist for prostate cancer PET imaging: [^64^Cu]-DOTHA2-PEG-RM26. Nucl Med Biol.

[43] Mansi R, Wang X, Forrer F, Waser B, Cescato R, Graham K, Borkowski S, Reubi JC, Maecke HR (2011). Development of a potent DOTA-conjugated bombesin antagonist for targeting GRPr-positive tumours. Eur J Nucl Med Mol Imaging.

[44] Lutje S, Rijpkema M, Helfrich W (2014). Targeted radionuclide and fluorescence dual-modality imaging of cancer: preclinical advances and clinical translation. Mol Imaging Biol.

[45] Kaloudi A, Nock BA, Lymperis E, Valkema R, Krenning EP, de Jong M, Maina T (2016). Impact of clinically tested NEP/ACE inhibitors on tumor uptake of [^111^In-DOTA]MG11-first estimates for clinical translation. EJNMMI Res.

[46] Nieciecki M, Dobruch-Sobczak K, Wareluk P (2016). The role of ultrasound and lymphoscintigraphy in the assessment of axillary lymph nodes in patients with breast cancer. J Ultrason.

